# Supplementary material for: New Frontiers in Health Literacy: Using ChatGPT to Simplify Health Information for People in the Community
Source: J Gen Intern Med. 2023 Nov 8;39(4):573–7. doi: 10.1007/s11606-023-08469-w (PMC10973278; doi:10.1007/s11606-023-08469-w)
Supplement: Supplementary file 1 — Supplementary file1 (DOCX 32 KB) [file 11606_2023_8469_MOESM1_ESM.docx]

# Appendix 2. ChatGPT Prompt Development

**Prompt Objective**

To rewrite health information in plain language, in alignment with health literacy recommendations.

**Health Literacy Principles of Interest**

Principles are taken from the Universal Precautions Toolkit and Patient Education Materials Assessment Tool. These include the following:

1. Text uses plain language (use common, everyday language; avoid jargon; use the active voice where possible). Assessed objectively using the Sydney Health Literacy Lab (SHeLL) Health Literacy Editor:
   - Grade reading score: target grade 8.0 or lower; adequate: grade 10.0
   - Complex language: target <15%; adequate <20% (based on author expertise)
   - Passive voice: target: 0–1(9), Adequate: 2
2. Text directly addresses the reader.
3. Text defines medical terms and acronyms the first time they are used.

Each prompt was testing in a new chat window within ChatGPT. JA and OM generated two revised versions across 4 different texts (sciatica, dementia, leukaemia, malaria) for each prompt that was trialled. Prompts were also assessed in terms of retention of key messages, level of detail, accuracy, and introduction of new information.

## Observations—Prompt Categories

**References to age and school grade**

- Referring to an age bracket rather than a school grade typically returned a lower grade reading score.
- This also reduced complex language and typically removed all passive voice.
- Prompting for 11 years old produced more natural language/better responses than for younger, e.g. 10 years old and maintained most of the detail.
- Sometimes the revised text would reference age, e.g. ‘Tell an adult’ rather than ‘See your doctor’ and ‘people like you’ when referring to children aged <14 years.
- Issues with stigma in this phrasing.

**People with English as a second language/non-native English speakers**

- Referring to non-native English speakers (in itself) did not tend to reduce grade reading score, complex language, or passive voice. When combined with reference to age/school, these scores improved (grade reading score ~8–11, low complex language and low/zero passive voice).

**People with low literacy**

- Similar to the combination of age/school + English as a second language.
- Typically retained detail and language read well, tone of original text was retained.
- Occasionally text was addressing a child.

**Explicit instructions describing health literacy principles**

- Grade reading score tended to be high.
- Passive voice often not removed, even when this was an explicit instruction. Sometimes ‘active’ voice was interpreted by ChatGPT as being more conversational/informal.

**Simplified language**

- Grade reading score and complex language tended to be lower, with passive voice removed.
- Revisions retained detail and key messages.

## Prompt Shortlist

Prompt shortlist comprised the following:

1. Rewrite the text into simplified language that is easy to understand.
2. Rewrite the text so it is suitable for non-native English speakers and people with low literacy.
3. Rewrite the text for people with low literacy.

**Objective assessments of shortlist prompts**

JA and OM generated two revised versions across 4 different texts (sciatica, dementia, leukaemia, malaria) for each prompt that was trialled.

The tables below present the objective scores for grade reading score, complex language and passive voice. Coloured cells show the extent that the texts generated by ChatGPT met target health literacy recommendations. Cells that are green indicate at least one of the two texts had a score in the target range. Cells that are yellow indicate at least one of the two texts had a score in the adequate range. Option 3 ‘Rewrite the text for people with low literacy’ was selected for use in this study.

**Grade reading score**

|  | Sciatica | Dementia | Leukaemia | Malaria |
| --- | --- | --- | --- | --- |
| *Original* | ***13.4*** | ***15.8*** | ***12.7*** | ***13.7*** |
| Option 1 | 9.1–13.4 | 12.0–12.3 | 9.7–10.5 | 10.5–10.7 |
| Option 2 | 12.3–13.5 | 13.4–14.9 | 9.7–10.6 | 10.7–10.9 |
| Option 3 | 9.8–11.0 | 8.8–9.7 | 9.6–10.6 | 9.0–9.4 |

**Complex language**

|  | Sciatica | Dementia | Leukaemia | Malaria |
| --- | --- | --- | --- | --- |
| *Original* | ***23.2*** | ***32.0*** | ***30.8*** | ***43.4*** |
| Option 1 | 11.9–19.2 | 16.3–16.5 | 19.9–25.0 | 23.5–28.0 |
| Option 2 | 17.8–22.1 | 16.7–19.8 | 20.4–27.9 | 22.9–27.8 |
| Option 3 | 11.4–13.9 | 5.7–6.7 | 22.5–25.2 | 17.2–18.3 |

**Passive voice**

|  | Sciatica | Dementia | Leukaemia | Malaria |
| --- | --- | --- | --- | --- |
| *Original* | ***4*** | ***2*** | ***2*** | ***3*** |
| Option 1 | 0 | 0–1 | 1–2 | 0–1 |
| Option 2 | 1–4 | 0–1 | 1 | 0–2 |
| Option 3 | 0–1 | 0 | 0–2 | 0 |

**References**

1. Brega A, Barnard J, Mabachi N, et al. AHRQ Health Literacy Universal Precautions Toolkit, 2nd Edition. Agency for Healthcare Research and Quality. Updated February 2015. Accessed 14 June 2017, http://www.ahrq.gov/professionals/quality-patient-safety/quality-resources/tools/literacy-toolkit/healthlittoolkit2.html

2. Shoemaker SJ, Wolf MS, Brach C. Development of the Patient Education Materials Assessment Tool (PEMAT): a new measure of understandability and actionability for print and audiovisual patient information. Patient Educ Couns. Sep 2014;96(3):395-403. doi:10.1016/j.pec.2014.05.027

3. Clinical Excellence Commission. NSW Health Literacy Framework. 2019-2024. Clinical Excellence Commission. Accessed 20 April 2022, https://www.cec.health.nsw.gov.au/__data/assets/pdf_file/0008/487169/NSW-Health-Literacy-Framework-2019-2024.pdf

# Appendix 3. Example Original Text: Eating Disorders

This excerpt is 398 words, Grade 14.2, with 20.2% complex language, and 3 passive voice constructions.

| Eating disorders are serious conditions related to persistent eating behaviors that negatively impact your health, your emotions and your ability to function in important areas of life. The most common eating disorders are anorexia nervosa, bulimia nervosa and binge-eating disorder. Most eating disorders involve focusing too much on your weight, body shape and food, leading to dangerous eating behaviors. These behaviors can significantly impact your body's ability to get appropriate nutrition. Eating disorders can harm the heart, digestive system, bones, and teeth and mouth, and lead to other diseases. Eating disorders often develop in the teen and young adult years, although they can develop at other ages. With treatment, you can return to healthier eating habits and sometimes reverse serious complications caused by the eating disorder.    Anorexia (an-o-REK-see-uh), also called anorexia nervosa, can be a life-threatening eating disorder. It includes an unhealthy low body weight, intense fear of gaining weight, and a view of weight and shape that is not realistic. Anorexia often involves using extreme efforts to control weight and shape, which often seriously interfere with health and daily life.  Anorexia may include severely limiting calories or cutting out certain kinds of foods or food groups. It may involve other methods to lose weight, such as exercising too much, using laxatives or diet aids, or vomiting after eating. Efforts to reduce weight can cause severe health problems, even for those who continue eating throughout the day or whose weight isn't extremely low.  Bulimia (buh-LEE-me-uh), also called bulimia nervosa, is a serious, sometimes life-threatening eating disorder. Bulimia includes episodes of bingeing, commonly followed by episodes of purging. Sometimes bulimia also includes severely limiting eating for periods of time. This often leads to stronger urges to binge eat and then purge.  Bingeing involves eating food — sometimes an extremely large amount — in a short period of time. During bingeing, people feel like they have no control over their eating and that they can't stop. After eating, due to guilt, shame or an intense fear of weight gain, purging is done to get rid of calories. Purging can include vomiting, exercising too much, not eating for a period of time, or using other methods, such as taking laxatives. Some people change medicine doses, such as changing insulin amounts, to try to lose weight.  Bulimia also involves being preoccupied with weight and body shape, with severe and harsh self-judgment of personal appearance. |
| --- |

*Source:* Mayo Clinic (<https://www.mayoclinic.org/>).

## Key messages in original text:

1. Eating disorders are serious conditions related to persistent eating behaviours that negatively impact physical and mental health.
2. Eating disorders often develop in teen and young adult years.
3. People with anorexia nervosa use extreme efforts to control their weight and shape.
4. Anorexia nervosa and bulimia can be life-threatening if left untreated.
5. Bulimia nervosa includes episodes of bingeing, commonly followed by episodes of purging.
6. Bingeing involves eating (often a large) amount of food in a short period of time, with a feeling of lack of control.

## Example 1 revised text—all key messages retained

The revised text was 275 words, Grade 10.8, with 9.1% complex language, and 1 passive voice construction.

| Eating disorders are when someone has serious problems with the way they eat that can hurt their health and life. The most common kinds are anorexia nervosa, bulimia nervosa, and binge-eating disorder. Eating disorders usually happen when people care too much about their weight, shape, and food and do things that can be harmful. This can make it hard for them to get the right nutrition, which can hurt their heart, digestion, bones, teeth, and other parts of their body. Eating disorders often start in the teenage and young adult years, but can happen at any age. Treatment can help people get better and stop the problems caused by their eating disorder.  Anorexia is an eating disorder that can be life-threatening. People with anorexia weigh too little and are very scared of gaining weight. They may do extreme things to control their weight, which can really hurt their health and daily life. For example, they may not eat enough, exercise too much, or make themselves vomit.  Bulimia is a serious eating disorder that can be life-threatening. People with bulimia have episodes where they eat a lot of food in a short time and feel like they can't stop. After they eat, they may feel guilty or afraid of gaining weight, so they do things like make themselves vomit or exercise too much to get rid of the food.  Bulimia can also mean that someone doesn't eat enough food for a while. This can make them want to binge eat even more, and then they will purge again. People with bulimia are also very worried about their weight and shape and often think badly about themselves. |
| --- |

All key messages were retained (100% across both raters)

| **#** | **Key message** | **Rater 1** | **Rater 2** |
| --- | --- | --- | --- |
| 1 | Eating disorders are serious conditions related to persistent eating behaviours that negatively impact physical and mental health. | ✓ | ✓ |
| 2 | Eating disorders often develop in teen and young adult years. | ✓ | ✓ |
| 3 | People with anorexia nervosa use extreme efforts to control their weight and shape. | ✓ | ✓ |
| 4 | Anorexia nervosa and bulimia can be life-threatening if left untreated | ✓ | ✓ |
| 5 | Bulimia nervosa includes episodes of bingeing, commonly followed by episodes of purging. | ✓ | ✓ |
| 6 | Bingeing involves eating (often a large) amount of food in a short period of time, with a feeling of lack of control. | ✓ | ✓ |

## Example 2 revised text—75% of key messages retained

The revised text was 224 words, Grade 12.3, with 11.1% complex language, and 1 passive voice construction.

| Eating disorders are serious conditions that can hurt your health and ability to do things you need to do. They often involve focusing too much on your weight, body shape, and food. The most common eating disorders are anorexia nervosa, bulimia nervosa, and binge-eating disorder. These disorders can harm your body, causing problems with your heart, bones, teeth, and digestive system. Eating disorders often start when people are young, but they can start at any age. With help, you can get better and start to eat healthier again.  Anorexia nervosa is an eating disorder that can be very dangerous. People with anorexia nervosa have a very low body weight, are afraid of gaining weight, and see themselves as bigger than they really are. They often try extreme things to lose weight, like not eating enough, exercising too much, or making themselves vomit. This can cause serious health problems, even if they are still eating normally throughout the day.  Bulimia nervosa is another serious eating disorder. People with bulimia often have episodes of binge eating, where they eat a lot of food very quickly and feel out of control. Afterward, they feel guilty and try to get rid of the calories by making themselves vomit or using other methods like laxatives. People with bulimia also worry a lot about their weight and how they look. |
| --- |

On average across both raters, 75% of key messages were retained

| **#** | **Key message** | **Rater 1** | **Rater 2** |
| --- | --- | --- | --- |
| 1 | Eating disorders are serious conditions related to persistent eating behaviours that negatively impact physical and mental health. | ✓ | ✓ |
| 2 | Eating disorders often develop in teen and young adult years. | ✓ | X |
| 3 | People with anorexia nervosa use extreme efforts to control their weight and shape. | ✓ | ✓ |
| 4 | Anorexia nervosa and bulimia can be life-threatening if left untreated | X | X |
| 5 | Bulimia nervosa includes episodes of bingeing, commonly followed by episodes of purging. | ✓ | ✓ |
| 6 | Bingeing involves eating (often a large) amount of food in a short period of time, with a feeling of lack of control. | ✓ | ✓ |
